# Restoring trust in public health: communications lessons from person-centered health care

**DOI:** 10.1093/eurpub/ckae151

**Published:** 2025-03-25

**Authors:** Robert Steiner, Seema Yasmin

**Affiliations:** Dalla Lana School of Public Health Sciences, University of Toronto, Canada; School of Medicine, Stanford University, CA, United States

Public health leaders in many settings habitually assume a command-and-control approach to crisis and emergency communications, in which public health authorities attempt to dominate a one-way “conversation” with a monolithic public.

That approach may build trust during a short, well-understood emergency. But the COVID-19 pandemic revealed that it does not build trust through situations that [1] are chronic [2], poorly understood, and [3] impact society beyond the expertise of public health leaders. In these circumstances, “command-and-control” communications can actually alienate the public.

Public health leaders can regain trust by applying three principles of person-centered health care to community engagement: first, by listening to the community and integrating listening data into public health decisions. Second, through rigorous transparency about the scientific tradeoffs that inform policies. Third, by tailoring communications to the distinct needs of different communities. The WHO and International Federation of Red Crescent and Red Cross, among others, insist on community engagement in public health communications. But during crises, public health leaders actually seem to lag their clinical medicine colleagues in precisely such two-way communications.

## The limits of command-and-control communications

Public health leaders around the world have long clung to three communications values the US CDC prints on wallet cards available on its website: “Be First”/“Be Right”/“Be Credible.” These principles are appropriate for localized, well-understood and short-term crises; the public-health equivalent of how an emergency-room physician might communicate while leading a life-saving intervention.

But the same three values can create “distrust” during a prolonged, novel public health threat. “Being First” tempts public health leaders to sideline other community leaders with greater legitimacy in their areas of expertise. “Being Right” means public health leaders might leave no space for the evolution of knowledge. “Being Credible” (combined with “Being First”) implies that public health leaders, by definition, have greater expertise than they actually do in the wide range of fields that come into play during an extended public health crisis, such as economics, education, labor, and human rights law.

This trifecta sets up public health agencies and leaders for failure. Even scientifically minded people sour on the façade of scientific omniscience. If public health leaders greet that skepticism by doubling down on the US CDC’s command-and-control formula, the resulting polarization will likely compound the dangers of the public health crisis itself.

To be sure, the CDC’s framework also urges “statements of empathy” [1]. But empathy without a rigorous understanding and respect for a community’s changing experience is merely performative.

## Person-centered care has lessons for public health communications

The World Health Organization’s Joint External Evaluation Tool measures members’ ability to “gather information on (public) perceptions, risky behaviors and misinformation” [2]. Rather than developing a deep understanding of their publics during a crisis, public health leaders often rush to communicate in one direction: through press conferences, websites, and social media. Person-centered clinical care, on the other hand, “encourages the active collaboration and shared decision-making between patients, families, and providers to design and manage a customized and comprehensive care plan” [3]. That model, which has long been accepted in medicine, offers three lessons that public health leaders can use to engage communities.

### Lesson 1. Generate listening data

In a person-centered, clinical setting, physicians and others on the care team begin by listening to the patient’s own understanding of their wellbeing. Similarly, the public health team can actually “measure” a community’s changing experience and values during an extended public health emergency. South Africa generated such data during the height of their COVID crisis. Local public health officers completed a simple, weekly “Social Listening Report” based on grassroots community interactions and submitted it to higher authorities, who used it for policy making and shared it publicly [4]. Aggregated and longitudinal data from grassroots listening complemented South Africa’s social media listening programs to measure changes in public sentiment more precisely than online listening could do alone, overweighted as it is to those who are most active on social media. Indeed, local social listening reports helped inform many of South Africa’s pandemic strategies (Motalatale Modiba, Chief Director: Communication, Gauteng Department of Health, South Africa, email communication, 22 February 2022).

### Lesson 2. Be transparent

Transparency is also key to building and retaining trust through an evolving, chronic public health crisis. When a healthcare team understands a patient’s concerns and perspectives, it can be transparent about trade-offs in healthcare decisions. Similarly, a public health team that understands community sentiment can be transparent about policy tradeoffs—acknowledging the limits of science and the legitimacy of changing values in different communities.

In democracies, public health leaders share decision-making with elected leaders. Political polarization makes the rigorous measurement of public health sentiment especially valuable as a bridge to more open-minded elected leaders who sometimes occupy swing positions of power.

### Lesson 3. Be accessible

Access to information is a powerful but poorly understood social determinant of health. Studies of US communities during the first 6 months of the pandemic suggest that exposure to misleading TV “news” was associated with higher rates of COVID-19 morbidity and mortality and lower adherence to public health measures [5].

A person-centered healthcare team adapts its communications to a patient’s unique needs and communications style. Public health leaders should use social listening reports to understand their populations’ unique informational needs. Using those data, they can design a portfolio of communications channels that include networks of trusted messengers, and existing media platforms with the established power to engage especially vulnerable communities.

## Listening in a public health crisis: two fast steps

Agencies can build a public sentiment-gathering tool modeled on South Africa’s District Social Listening Reports, for use in crisis. The tool should be very easy for local public health officers to complete, consistent enough across regions and time to detect trends, and gather data that are immediately relevant to public health decision-making. South Africa’s experience echoes guidelines from the International Federation of Red Cross and Red Crescent Societies on how to build community feedback mechanisms. These guidelines emphasize that data collection should be light-touch and include a minimal number of questions which are asked on a regular basis [6].

Public health agencies should also map the information ecosystems of their communities to pinpoint subgroups that are especially vulnerable to false health information. In the USA, an Information Inequity Index and Information Ecosystem Map are now under development for this purpose. The project measures the density of local newspapers and access to credible, fact-checked TV and radio news; broadband and mobile network coverage; and literacy levels and languages spoken on a community level as well as literacy and language-appropriate access to news in communities. These data are being correlated against healthcare outcome data such as vaccination status, adherence to non-pharmaceutical interventions (NPIs), and trust in government and scientific institutions.

If, having learned from COVID, public health aims to share responsibility with the community, public health leaders should listen to their communities as accurately as person-centered physicians listen to their patients.

Conflict of interest: None declared.
